# Unfolding newer innovations for tomorrow’s emergencies and disasters 2024 (UNITED’24): international disaster medicine conference in Kerala, India

**DOI:** 10.3389/fpubh.2026.1656383

**Published:** 2026-03-06

**Authors:** Kelsey G. Trulik, Jiju Joseph, Trini A. Mathew, Vishnu Menon, Keith J. Alangaden, Shammy D. Lambert, Jeffy C. Jacob, Jobin J. Thrickoikal, Marc Rosenthal, Gilbert M. Burnham, Vijaya A. Kumar

**Affiliations:** 1 Department of Emergency Medicine, Wayne State University, Detroit, MI, United States; 2 Department of Emergency Medicine, Believers Church Medical College Hospital, Thiruvalla, Kerala, India; 3 Department of Internal Medicine, Center for Emerging and Infectious Diseases, Wayne State University, Detroit, MI, United States; 4 Department of Emergency Medicine, National Health Service, London, England, United Kingdom; 5 Department of International Health, The Johns Hopkins Bloomberg School of Public Health, Baltimore, MD, United States

**Keywords:** disaster medicine, international conference, medical ethics, resilience, mock drill

## Abstract

**Introduction:**

Kerala faces numerous disasters and has disaster management teams at all levels. However, efforts are needed to train professional staff and educate the general population for effective disaster response.

**Methods:**

The Unfolding Newer Innovations for Tomorrow’s Emergencies and Disasters (UNITED’24) conference was hosted by Believers Church Medical College Hospital (BCMCH) in Kerala, India in November 2024. The conference on disaster preparedness included pre-conference asynchronous lectures, and in-person didactics, panels, exercises, and a mock drill.

**Results:**

Of the 434 conference attendees, 323 completed the anonymous post conference survey. Respondents were predominantly between 22 and 29 years-old (54%), female (68%), from Kerala (97%), and medical or nursing students and residents (75%). The top five most informative sessions identified by the respondents were on disaster planning and response (57%), triage ethics (51%), building resilience post-disasters (50%), climate change (35%), and the disaster mock drill (32%).

**Discussion:**

This first international disaster medicine conference in India trained multidisciplinary professionals in disaster management and facilitated development of a disaster response center in Kerala through strategic academic and government partnerships contributing to a more disaster-resilient India.

## Introduction

Kerala, with a population of roughly 35 million people, ranks as the 8th most densely populated state in India (1). It is especially vulnerable to both natural and anthropogenic disasters, including coastal erosion, landslides, floods, and droughts. Its exposed location is particularly susceptible to the increasing impact of global warming and climate change (2).

### Kerala’s disaster timeline

Major disasters in Kerala since 2015 include the Puttingal temple fire (2016), Cyclone Ockhi (2017), the catastrophic floods of 2018 and 2019, and the Wayanad landslides in 2024. From 2015 to 2022, Kerala reported 2,239 landslides—representing nearly two-thirds of all landslides documented across India during that period (3). According to the 2023 Landslide Atlas of India, Kerala’s soil, particularly in Wayanad, is extremely vulnerable to landslides due to the slope of the terrain and the heavy amount of rainfall (4).

During the 2018 monsoon season, Kerala experienced the worst floods since 1924 (5). Unprecedented rainfall caused widespread flooding and landslides. The state recorded a rainfall excess of 96% (6). Approximately 5.4 million people were impacted by the floods, and 1.4 million people were displaced. There were 433 documented fatalities (5). More than 10,000 homes were destroyed and another 111,000 were severely damaged. The floods extensively damaged 86,850 hectares of agricultural land and 3,000 hectares of fish farms. Tens of thousands of kilometers of roads were damaged. An estimated $US 192 million were spent on medical bills, repairing housing and agricultural damages, animal carcass removal, infrastructure restoration, and debris removal (6, 7). In response to flooding, the Kerala government quickly mobilized rescue and relief operations, involving national agencies including the National Disaster Response Force (NDRF), Indian Army, Navy, Air Force, and Coast Guard. Despite proactive steps, inadequacies in rainfall forecasting impeded accurate monitoring, warning, and response. The 2018 floods highlighted deficiencies in the existing preparedness, such as terrain management, slope stability assessment, and flood drainage plans, emphasizing the need for improved preventive planning and infrastructure resilience (6, 7).

In August 2019, Kerala recorded 123% of its normal average rainfall. The peak rainfall period saw a 394% increase from normal, significantly contributing to widespread flooding and landslides across multiple districts. There were 125 fatalities, with significant damages and destruction of homes. The floods extensively damaged 6,244 hectares of agricultural land and killed 460,350 livestock. Thousands of kilometers of roads were damaged. An estimated $US 133 million were spent on repairing housing and agricultural damages, animal carcass removal, infrastructure restoration, and debris removal (8).

The Meppadi Landslide occurred in the Wayanad district on July 30, 2024, triggered by extreme rainfall. This disaster affected four settlements, injuring 378 persons, causing 231 confirmed deaths and destroying approximately 1,500 houses. Approximately 626 hectares of crops were destroyed with additional damages to other critical infrastructure. Immediate relief operations deployed approximately 5,000 individuals. An estimated $US 74 million were spent on medical bills, immediate relief operations, and infrastructure restoration (9).

The response by the NDRF, Indian Army, and Air Force was prompt. However, the scale of destruction highlighted gaps in disaster mitigation planning including dissemination of early warning. These disasters indicate a clear need for improved local and national coordination, organization, and planning for flood preparedness (8, 9).

### India’s disaster organizations

The Disaster Management Act of 2005 established the complementary National Disaster Management Authority (NDMA) and NDRF to effectively prepare for, manage, and respond to both man-made and natural disasters in India (10). The NDMA is the policy-making and strategic body of disaster management in India. It is responsible for developing national policies and guidelines for disaster management and overseeing enforcement and implementation of these policies across the country (11). The NDRF is a specialized, on-ground response force. They provide immediate and direct action in the face of disaster, carrying out search, rescue, and relief operations. The NDRF is also responsible for training community volunteers and local agencies in disaster rescue and response through regular mock drills and exercises (12). Both the NDMA and NDRF aim to build a safer, better-prepared, and disaster-resilient India through prevention, mitigation, preparedness, and prompt response to disasters (11–13).

The Kerala State Disaster Management Authority (KSDMA) is responsible for formulating policies, plans, and guidelines for disaster risk reduction and management in the state. It coordinates with district authorities and various government departments to ensure effective preparedness, mitigation, response, and recovery measures. KSDMA also facilitates capacity building, public awareness campaigns, early warning systems, and scientific research to enhance community resilience and minimize disaster impact (14). Each district in Kerala has its own District Disaster Management Authority (DDMA), responsible for planning, coordinating, and implementing disaster management activities at the district level (15). The State Emergency Operations Centre (SEOC) functions as the primary coordinating authority during disasters. It supports the State Relief Commissioner in managing disaster response and recovery operations (16). The District Emergency Operations Centres (DEOCs) provide continuous support during disasters: coordination with local agencies, data collection, and assistance in implementing disaster management plans (17). All these regulatory bodies work collaboratively to enhance Kerala’s resilience to disasters.

### Why host an international disaster conference?

The recent history of repeated natural and man-made disasters in Kerala highlights critical gaps in disaster preparedness, public awareness, regional response capabilities, and inter-agency coordination. Recognizing this, we initiated the UNITED’24 International Conference, held from November 14–17, 2024. A key objective of UNITED’24 was to initiate a capacity-building process by fostering strong collaboration between government bodies, including the Pathanamthitta District Administration, DDMA, KSDMA, and BCMCH, a private healthcare and academic institution located in Thiruvalla, Kerala (13). Through UNITED’24, BCMCH aims to be a proactive leader in safeguarding communities by promoting resilience, preparedness, and collaboration in disaster mitigation.

In this manuscript, we describe the processes for collaboration and establishing an international disaster conference. We summarized the discussions and provided a list of suggestions and future plans that can be adopted by other organizations and regions to improve disaster response and management. We provide suggestions based on local and international experiences and expertise, as well as responses from the participants.

## Materials and methods

### Planning

Planning for the conference began in April 2023 between leaders at BCMCH in Thiruvalla, Kerala, India and Wayne State University (WSU) in Detroit, Michigan, USA. Over the following months, the organizing teams developed collaborations with faculty of Johns Hopkins University (JHU) in Baltimore, Maryland, USA, and George Washington University (GWU) in Washington DC, USA.

Seven primary objectives were developed based on needs identified by the local planning committee to improve disaster medicine in Kerala:

1. Share effective disaster response strategies and resilience-building techniques.
2. Strengthen international partnerships.
3. Improve disaster preparedness, response, and recovery policies.
4. Promote innovative technologies and research in disaster management.
5. Raise public awareness about disaster preparedness.
6. Address the local impact of global climate change.
7. Provide capacity-building and disaster training sessions.

This first conference was intended for a broad audience, including medical and nursing faculty and students, paramedical staff, hospital administrators, federal and state government employees, school and college students, and community members with an interest in disaster preparedness. Speakers were selected based on their expertise, relevance to the conference themes, availability, and alignment with the stated objectives. Coursework from the Health Emergencies in Large Populations (HELP) program—developed by the JHU Bloomberg School of Public Health—was integrated into the event to train participants in the public health principles of disaster preparedness and management.

### Pre-conference

BCMCH hosted three virtual webinars in October 2024 leading up to the conference. The webinar Zoom links were shared with all registered conference participants and on various social media platforms. There were four lectures at each webinar, led by distinct experts in the field from both India and the United States. Session topics included the *Need for a Disaster Medicine Conference, Wayanad Experience, Nuclear and Radiological Disasters, Traumatic and Explosive Events, Disaster and MSF Experience in Iraq, Disaster Definition, Disaster Dynamics, Pre-Disaster Paradigm, Disaster—Current Scenario and National/Local Disaster Guidelines and Management, SALT* (*Sort, Assess, Lifesaving interventions, Treatment/transport*) *Triage and Government Programs, Critical Role of Mock Drills in Disaster Management and Preparedness, Biological and Chemical Disasters, Disaster Decontamination and PPE (Personal Protective Equipment), HELP—Disaster Workshop.*

In parallel, the organizing team developed an asynchronous learning platform that offered conference attendees access to pre-recorded videos and reading materials from the HELP curriculum. Module topics included: *Understanding Disasters and Disaster Assistance*, D*isasters from Natural Hazards, Structure of International Assistance, Planning for Disasters, International Health Regulations, Ethics, Delivery of Health Services, Disease Management - Communicable and Non-Communicable Diseases, Water, Sanitation, and Hygiene, Mental Health, and Palliative Care*. All modules included research articles and pre-recorded videos provided by the HELP course.

### Conference

The in-person conference spanned four days and featured didactic sessions, a full-scale disaster mock drill, and a consensus meeting of regional experts. Didactics included lectures, group exercises, panel discussions, and video-based learning (Appendix 1). A blend of passive and interactive formats were used to enhance engagement. Attendees who completed the program received a certificate from the iHELP course.

### Mock drill

The capstone of the conference was a half day disaster mock drill planned by WSU and BCMCH faculty, as well as local stakeholders including local police and fire departments, EMS agencies, the NDRF, the Thiruvalla Health Department, and Abda Mitra Volunteers. The scenario simulated a building collapse involving extrication, field triage, transport, emergency department (ED) care, and follow-up treatment. Participants included hospital staff, emergency medicine faculty, conference attendees, and local authorities. They were assigned to specific sites including: the location of the building collapse for immediate recovery and triaging, the ambulance bay of the nearby hospital (BCMCH) for reassessment, various treatment stations throughout the hospital (based on severity of triage assessment) for patient care, and the command center for communication and oversight. Attendees who did not participate in the mock drill watched a video of the sequence of events after it concluded. All participants then gathered for a structured debriefing session to discuss successes, challenges, and opportunities for improvement.

### Consensus meeting

The UNITED’24 organizing committee organized a consensus conference to facilitate dialogue between stakeholders regarding the current state and future of disaster management in Kerala, India. The planning phase was done to identify five specific groups that would provide diverse perspective and expertise, which included:

1. Hospital Administrators and CEOs
2. Clinical Response Teams
3. Local Government Officials
4. Wayanad 37 Response Team
5. Prominent Community Leaders and NGOs

Five to seven subject-matter experts were selected for each group by local physicians and organizers based on community feedback and established regional leadership positions. An expert consensus meeting using structured, open-ended prompts was used to identify key gaps in current disaster systems and develop priorities for establishing a disaster response hub in Kerala. Groups met individually for about one hour. Each group had one US-based physician facilitator and a local emergency medicine resident as a scribe. Groups subsequently completed an anonymous poll to select the top 10 of 30 topics essential to meeting the UNITED’24 objectives. These topics were synthesized from group discussions and conference goals.

### Post-conference survey

A 42-question descriptive cross-sectional post-conference survey was developed by the UNITED’24 team using Qualtrics software (Qualtrics, Provo, UT) (18), incorporating a mix of multiple-choice, Likert scale, and open-ended responses. The survey covered a range of topics, including attendee demographics, professional background, conference objectives, venue and facilities, session topics, speakers, group exercises, the mock drill, application of learning, overall experience, and areas for improvement. A QR code with access to the survey was projected on all screens on the final afternoon of the conference and attendees were asked to complete the survey prior to leaving. The first question screened for age; attendees under 18 years of age were excluded from further participation. A qualitative analysis was performed of the available responses obtained from participants. As this was a convenience sampling, there was some missing data from the survey, which is a limitation of this type of survey.

We received a determination from the Wayne State University Institutional Research Board in January 2025 stating that this project does not constitute human participant research and is thus exempt from IRB review.

## Results

### Pre-conference

**Figure 1:**
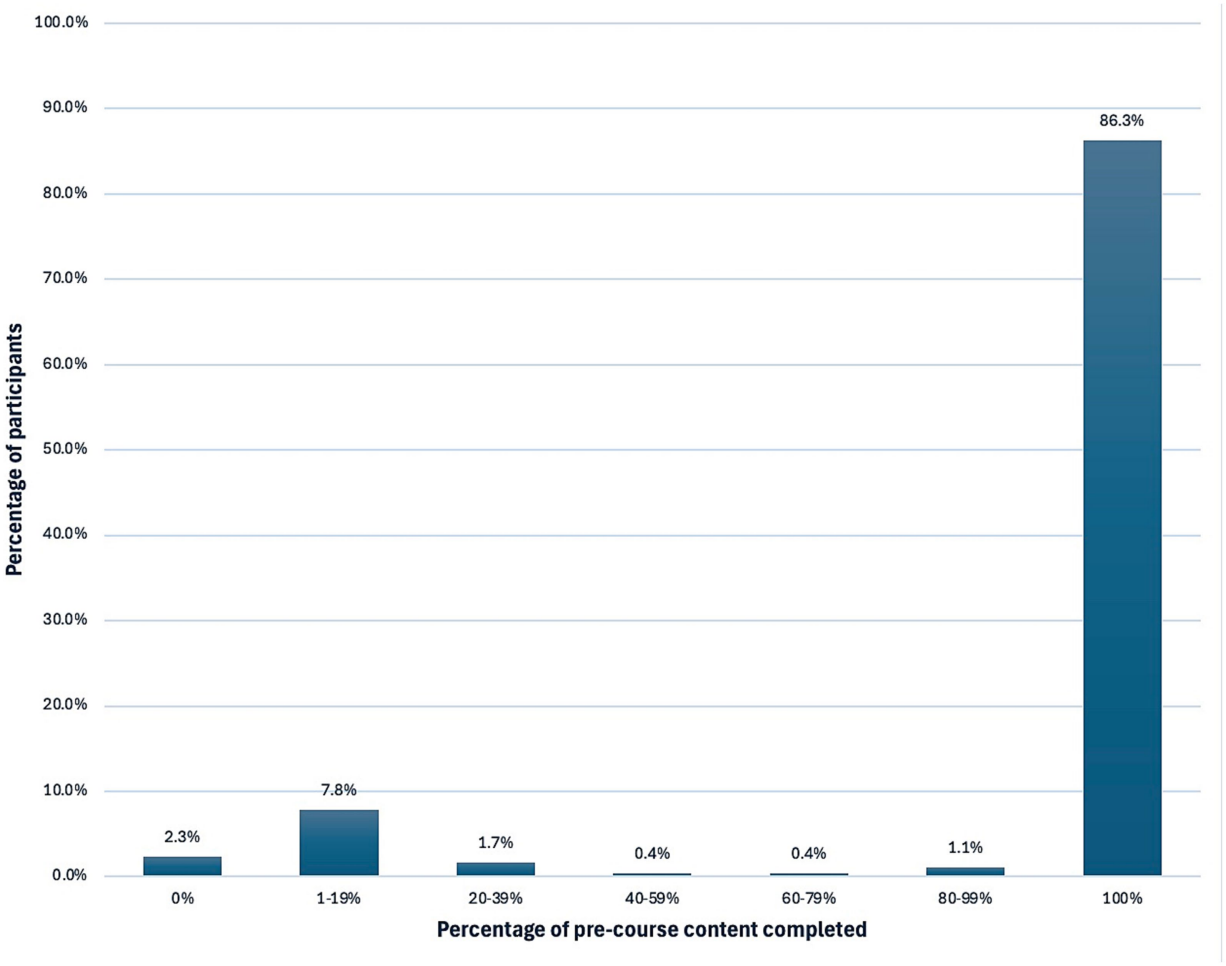
Proportion of registrants who completed different percentages of the pre-course content (*n* = 475).

A total of 475 people registered for the UNITED’24: Integrated HELP pre-conference course. 89% of participants completed the entire course. Registrants spent on average, 351 minutes engaging with pre-course material. Figure 1 shows the proportion of registrants who completed different percentages of the course content. An average of 70 registrants attended each virtual webinar session, with the majority being students.

### Post-conference

A total of 434 people registered for and attended the UNITED’24 conference. Of these, 323 completed the post-conference survey via Qualtrics. The demographics of the attendees is outlined in Table 1. Most participants (80%) felt the conference topics were very relevant to their work and/or interests, and 82% thought the speakers were very effective in engaging the audience and presenting complex information clearly. Conference attendees participated in two group exercises created by the HELP course - Post-Disaster Damage & Needs Assessment and Establishing Health Services. Participants thought these exercises were strongly beneficial (73%) and allowed for good discussion and thinking (67%). The overall selection of conference topics and content was widely recognized as being the most valuable aspect of the conference (26%). The top five-rated most informative and useful conference sessions were Disaster Definitions, Disaster Cycle, Disaster Planning, and Disaster Response (57%), Emergency Disaster Triage Ethics During Mass Casualty (51%), Building (Individual) Resilience in the Aftermath of Disasters (50%), Climate Change Impacts and Health Risks: Science, Mitigation, and Adaptation (35%), and Disaster Mass Casualty Mock Drill (32%). The session on Emergency Disaster Triage Ethics During Mass Casualty began with a participant poll. Participants were asked to respond to the question “What do you think is the biggest ethical issue in a disaster?” with a single word. A total of 294 attendees participated. The audience identified the top five ethical issues in disaster settings as the allocation and distribution of resources, triage decision-making, ensuring equitable care, and addressing the needs of vulnerable populations.

**Table 1:** Demographics of conference participants.

Characteristic	Category	Frequency	Percentage
Age (*n* = 323)	<18	7	2%
	18–21	94	29%
	22–29	176	54%
	30–39	28	9%
	40–49	9	3%
	50–59	4	1%
	60–69	5	2%
Gender (*n* = 313)	Male	100	32%
	Female	213	68%
Educational status (*n* = 306)	MBBS student	83	27%
	Nursing student	86	28%
	Intern	19	6%
	Nurse	4	1%
	Resident	44	14%
	Physician/specialist	30	10%
	Paramedic	18	6%
	Social worker	12	4%
	Other	10	3%
Medical specialty (*n* = 59)	Emergency medicine	25	42%
	Non-Emergency medicine	34	58%
Institution setting (*n* = 307)	Private	282	92%
	Government	21	7%
	Academic/university	280	91%
	Non-university	23	7%

The mock drill received positive reviews, with 22% of attendees rating it as the most valuable and impactful session of the conference. Most participants (>85%) rated the drill’s organization as good or excellent, found the drill realistic, felt positively challenged, and reported improved understanding of emergency protocols. The triage training was frequently praised (24%), as was the opportunity to enhance quick decision-making skills, and learn about, setting up, and managing an Emergency Operations Center (EOC). A few participants appreciated the guidance provided by experienced professionals, such as NDRF officers and international experts, as well as the post-drill debriefings that reinforced key lessons and identified areas for improvement. A total of 89% of the attendees completed the workshop stating that they had improved confidence in following disaster protocols.

Roughly one-third of attendees reported wanting more hands-on participation, improved clarity for observers, and better overall communication during the mock drill. They suggested focusing on enhanced communication and coordination among teams, as well as ensuring roles and responsibilities are clearly defined and effectively communicated (29%). Attendees suggested several improvements such as more hands-on activities for observers rather than passive observation (27%), equipping the site with sufficient first aid kits, medical supplies, and modern technological tools (9%), use of live broadcasts or more designated observation areas (7%).

Participants also highlighted the group exercises (17%), expert speakers with specific note to international faculty (13%), and panel discussions (7%) as valuable in enhancing their understanding of disaster management. Networking opportunities, such as the chance to meet like-minded professionals (“meeting of the minds”), also stood out as valuable for fostering collaboration and exchanging ideas.

Many participants wanted more interactive sessions (38%), such as small group discussions, panel-audience interactions, and audience-speaker engagement. Others suggested making the four-day conference shorter (26%), diversifying topics (12%), avoiding repetitive material (9%), and limiting the use of virtual platforms. One session on “Orthosis and Prosthesis in Trauma and Disasters” was noted for having limited engagement by 17% of attendees, as the speaker joined via Zoom. Overall, attendees recommended shorter, more concise sessions with a balance between lectures and practical exercises to maintain engagement (8%). Networking opportunities among delegates and speakers were encouraged, along with addressing language barriers in multilingual audiences.

Suggested topics for future conferences include climate change (14%), and expanded discussions on mental health (14%), including post-disaster mental health support, stress management, and resilience-building techniques. Specialized areas such as triage, ethics in disaster management, support for vulnerable populations (ie. pediatrics, geriatrics, disabled), application of advanced technology, and the roles and responsibilities of specific groups during emergencies (ie. social workers and nurses) were also suggested for inclusion. Some attendees (8%) also wanted training in first response protocols - Basic Life Support (BLS), and Advanced Life Support (ALS).

We assessed participant agreement/disagreement with how well they felt the conference addressed the UNITED’24 learning objectives (Table 2). Participants chose to attend the conference for various reasons including educational growth (71%), networking and international exposure (9%), personal interest (8%), professional development (6%), interest in emergency medicine (4%), and recognition and certification (2%). Many attendees reported being likely to apply what they learned at the conference to their field of work (74%) and feeling inspired to pursue additional learning opportunities or certifications in disaster medicine (90%). Overall, the conference experience was rated as good to excellent by 98% of attendees, and 80% were interested in attending a future UNITED conference.

**Table 2:** Participant agreement with conference meeting stated objectives.

Objective	Strongly agree	Somewhat agree	Neither agree nor disagree	Somewhat disagree
The conference provided valuable opportunities for sharing effective disaster response strategies and resilience-building techniques	173 (62%)	96 (35%)	7 (3%)	2 (1%)
The conference facilitated valuable networking opportunities that could lead to future international partnerships	167 (60%)	94 (34%)	14 (5%)	3 (1%)
The policy discussions at the conference were relevant and provided actionable ideas for improving disaster response and preparedness in my region	167 (60%)	95 (34%)	15 (5%)	1 (0%)
The conference introduced innovative technologies and research findings that could enhance my professional disaster response capabilities	151 (54%)	108 (39%)	14 (5%)	5 (2%)
The conference emphasized the importance of public awareness in disaster preparedness and provided useful strategies for community engagement	177 (64%)	87 (31%)	12 (4%)	2 (1%)
The conference addressed global challenges such as climate change and pandemics in a way that felt relevant to my professional goals	176 (63%)	85 (31%)	14 (5%)	3 (1%)
The workshops and training sessions provided at the conference effectively enhanced my skills in disaster response	169 (61%)	95 (34%)	13 (5%)	1 (0%)

## Discussion

### UNITED’24 overview

UNITED’24 was a four-day disaster medicine and emergency preparedness conference, combining theoretical knowledge with interdisciplinary discussions and real-world applications. Recognizing that Kerala faces recurring disasters with clearly reported gaps in public awareness, disaster preparedness, and response efforts, conference organizers designed the event to directly address these challenges. The conference brought together over 400 participants and 30 speakers from various institutions across the world, emphasizing knowledge sharing and collaboration. The conference focused on approaches to disaster response and recovery through lectures, group and panel discussions, and case studies from local events like the Kerala floods and Nepal earthquake. The culminating large-scale simulation drill provided an accurate and hands-on example of the importance of emergency preparedness, coordinated efforts, efficiency, and practical execution of effective disaster management guidelines. The conference successfully increased awareness of disaster preparedness throughout Kerala and facilitated coordination between local and national organizations to strengthen response efforts.

### Limitations

There were several limitations in the design and implementation of the UNITED’24 post-conference survey. Firstly, though there were clear conference objectives, they did not translate to learning outcomes. Secondly, not all attendees completed the survey, which resulted in selection bias, incomplete data, and limited generalizability as participants were predominantly students. The length of the survey may have influenced the responses as there was a decrease in responses further along in the survey, as did the quality and detail of the written responses. Of note, there were also more responses related to the sessions in the initial days of the conference, which may be attributable to length of the conference and attendance fatigue. Additionally, no inferential tests were conducted since the subgroup analysis would not increase the value or change the outcome of our qualitative analysis among this cohort. The surveys contained many closed-ended questions which do not encapsulate the long-term aims of this conference and drive meaningful discussions for future directions in Kerala’s disaster management. This is short term evaluation and follow up studies are pending.

### UNITED’24 lessons learned

Conference participants mentioned areas to improve upon for future disaster management conferences, including more interactive experiences, diversified topics (role of nurses in disasters, impact of disasters on vulnerable populations, climate change, and technology), and networking opportunities. Overall, the conference received overwhelmingly positive results, with most attendees acknowledging improved understanding, confidence, and skills on disaster management.

### Kerala’s future directions

Disaster management efforts in India have long focused on response and recovery rather than preparedness, mitigation, and rehabilitation. The KSDMA has received national acclaim for their efficient and effective rescue and search operations (3). However, stronger government-led efforts are needed that emphasize community participation, drive sustainable developments, train and educate personnel, and implement innovative measures to drive improvements in disaster management (19).

Several central government guidelines outlining changes to improve disaster management have been proposed, including the 2016 10-point agenda on Disaster Risk Reduction and the NDMA landslide risk management plan (2019). However, both agendas remain theoretical, lacking proper implementation even at the local level (3, 19). This creates opportunities to lead efforts to improve disaster management. Several efforts have previously included addressing and combating climate change, diversifying the local economy, implementing a reliable early public warning system, funding structural interventions, ensuring infrastructure resilience, coordinating national and international efforts, and raising public awareness (3, 20–22).

Climate change is the leading cause of rising sea temperatures and annual rainfall, leaving Kerala vulnerable to natural disasters, including landslides, floods, and droughts (3, 20). Per the United Nations, the best way to understand disasters, and therefore be able to prepare for them, is to analyze, collect, and understand climate change risk-related data (20). Initial efforts must be taken to implement large scale vulnerability and climate change assessments to understand the situation and risks associated with the current environment (3, 21). This knowledge will allow local and national leaders to drive actionable change, motivating the development and implementation of renewable and sustainable practices.

Rural communities that rely on agricultural livelihoods are particularly vulnerable to the impacts of disasters. These communities become economically insecure when large volume crops are destroyed during disasters (3). Strong efforts are needed to diversify rural economies to stabilize livelihoods and combat the negative impacts from livelihood shocks.

While early warning systems exist in Kerala, as noted from the “orange alert” that was broadcasted during the Wayanad landslides, the system is oftentimes criticized for being delayed and unreliable, thus leaving communities uninformed and vulnerable (3, 21). Strong efforts must be placed to quickly disseminate accurate information to at-risk communities ahead of disaster impact to allow appropriate time to prepare and protect themselves.

Structural interventions such as slope stabilization and proper drainage systems have previously been proposed as potential risk-reduction strategies (3, 7–10). However, the implementation of these ideas remains a challenge in Kerala given the natural environment (3). The main problem facing vulnerable communities is the impacts from rapid succession of disasters. Proper rehabilitation measures are lacking. This highlights the importance of infrastructure resilience. This includes quickly building resilient roads, bridges, drainage systems, and other critical infrastructure that are more resistant to extreme weather events (23).

Kerala must partner with other national (Indian Red Cross Society, NDMA, NDRF, and non-governmental agencies), bilateral (neighboring country’s governments), and international organizations (United Nations and International Federation of Red Cross and Red Crescent Societies) to strengthen their coordinated efforts. These organizations offer unprecedented support, aid, and advice to not only respond to and recover from disasters, but also to build sustainable disaster plans (20). Finally, a nation is only as strong as its community. All efforts must be taken to raise public awareness surrounding disasters so that all citizens are informed about how to address future disasters when they strike (3).

### The role of BCMCH

The evident gaps in disaster preparedness and coordinated response underscore the urgent need for a centralized institution that can spearhead training, planning, and execution. BCMCH aspires to become a leading disaster response center in Kerala by transforming the lessons learned from recent regional disasters into actionable strategies. Through UNITED’24, BCMCH took a pioneering step by being the first institution in the country to bring together international disaster experts, national leadership, and government agencies such as the NDMA, NDRF, KSDMA, and DDMA under one collaborative platform. This initiative marked the beginning of BCMCH’s vision to function not just as a medical institution, but as the operational and academic nucleus of disaster response in the state. By continuously hosting simulation-based trainings, strategic planning workshops, and inter agency drills, and by fostering enduring partnerships with global and national leaders, BCMCH aims to build a robust, scalable disaster management ecosystem and provide real-time support during crises.

## Conclusion

Our initial efforts increased awareness of disaster preparedness amongst the participants. This year’s UNITED’24 conference served primarily as an awareness-building initiative and included a large cohort of students and faculty, particularly young medical professionals who represent the future of disaster medicine in India. We have presented the results of this workshop on several international Emergency Medicine platforms including the 2025 American College of Emergency Physicians (ACEP) Conference and the 2025 European Society of Emergency Medicine (EUSEM) Conference. We have started implementing streamlined, annual theme-based disaster conferences focused on key stakeholders who will be directly involved with disaster response. The UNITED’25 conference was well attended and planning has commenced for preparing BCMCH as a regional center for disaster preparedness, equipped with the information, support, resources, and network to function effectively and efficiently in times of crisis. These focused conferences will serve as platforms for bringing essential resources into the state, thereby enhancing overall disaster preparedness capabilities.

Through relationship-building strategies, BCMCH has positioned itself with state (KSMDA) and national (NDMA and NDRF) organizations to coordinate efforts locally and nationally. This strategic networking will improve disaster preparedness, response, and recovery with the shared goal of making India a more disaster-resilient nation. Additionally, we propose developing comprehensive training programs for faculty and students, which would create a sustainable pipeline of knowledgeable individuals ready to respond effectively during emergencies, engage with disaster medicine, ask critical questions, and get involved at the local level.

In summary, our experiences identified certain key aspects of disaster preparedness and response. Active participation by students and leaders of local communities helped identify gaps. We have provided solutions which may be of interest to the broader scientific communities (hospitals, NGOs, government agencies) to prepare and respond to national disasters.

## Data Availability

The raw data supporting the conclusions of this article will be made available by the authors, without undue reservation.
