# Supplementary figures and images for: Unfolding newer innovations for tomorrow’s emergencies and disasters 2024 (UNITED’24): international disaster medicine conference in Kerala, India
Source: Front Public Health. 2026 Mar 6;14:1656383. doi: 10.3389/fpubh.2026.1656383 (PMC13002826; doi:10.3389/fpubh.2026.1656383)

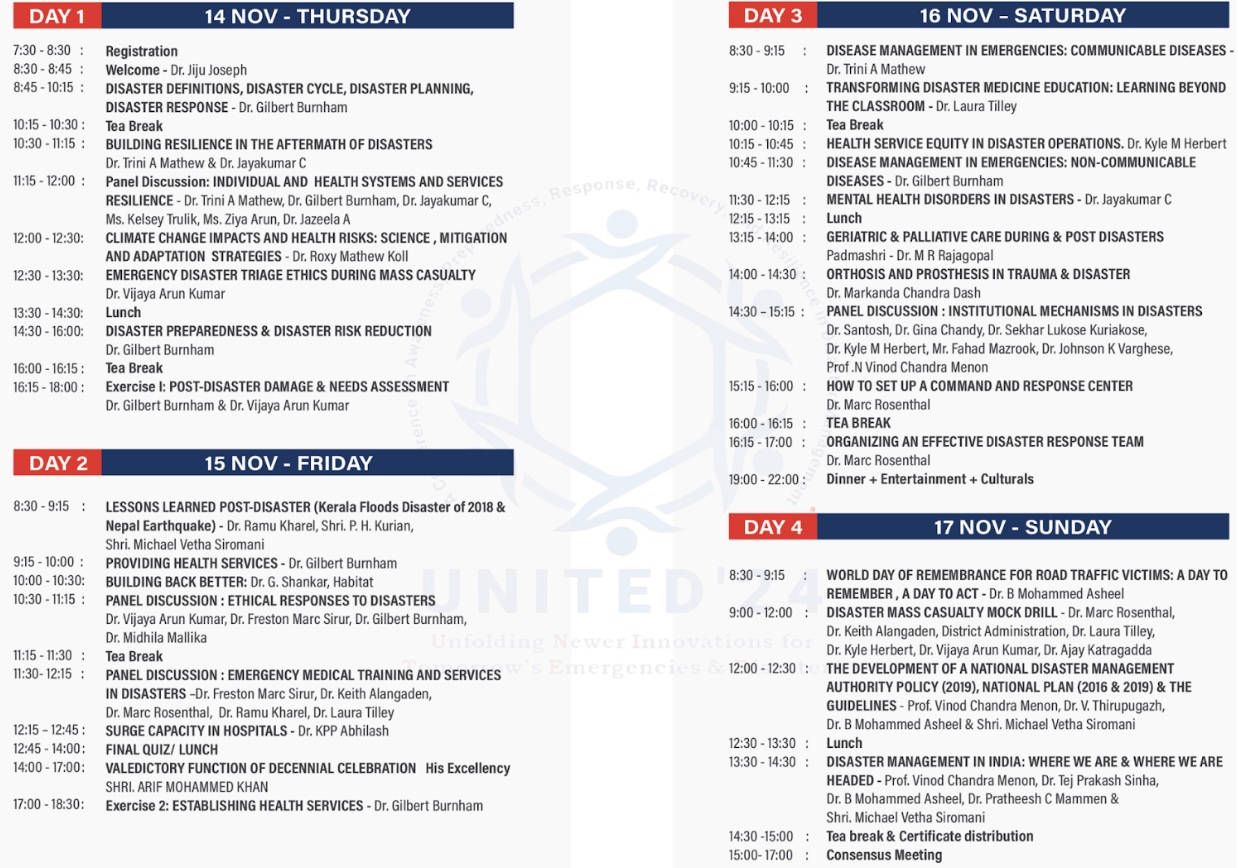

Supplement: Supplementary file 1 [file Image_1.JPEG]
